# Refractive outcome and visual quality of Ray-Tracing Guided LASIK: a prospective study

**DOI:** 10.1186/s12886-025-04517-7

**Published:** 2025-12-09

**Authors:** Yue Wang, Yuanyuan Zhang, Jiayu Pan, Jifan Wang, Xuejun Fang

**Affiliations:** 1 Aier Academy of Ophthalmology, Central South University, No.188, Furong South Road, Tianxin District, Changsha, Hunan 410004 PR China; 2 Liaoning Aier Eye Hospital, No. 99, Hepingbei Road, Heping District, Shenyang, PR China

**Keywords:** Ray-tracing, Refractive outcomes, Visual acuity, Aberrations, Spherical equivalent, Sightmap

## Abstract

**Purpose:**

This study aimed to evaluate the safety and effectiveness of Ray-Tracing Guided LASIK surgery and to compare the refractive outcomes and visual quality between patients with spherical equivalent (SEQ) differences of ≤ 0.75D and > 0.75D, as measured by the InnovEyes sightmap and subjective refraction.

**Design:**

Prospective, observational cohort study.

**Subjects:**

Forty-six patients (89 eyes) undergoing Ray-Tracing Guided LASIK with preoperative SEQ differences between sightmap and subjective refraction of either ≤ 0.75D or > 0.75D.

**Methods:**

Patients underwent preoperative examinations using InnovEyes sightmap diagnostic equipment to detect and measure biological parameters. A ray tracing-based algorithm was used to determine ablation contours, with direct application of ablation data for SEQ differences ≤ 0.75D and parameter adjustment for differences > 0.75D. Outcomes included uncorrected distance visual acuity (UDVA), corrected distance visual acuity (CDVA), manifest refraction spherical equivalent (MRSE), and total higher-order aberration (HOAs), measured preoperatively and six months postoperatively.

**Results:**

Six months post-surgery, 100% of eyes in both groups achieved a postoperative UDVA of at least 20/25, with 76% of the ≤ 0.75D group and 81% of > 0.75D reaching 20/16. Additionally, 33% and 16% reached 20/12.5 in these groups, respectively. Postoperative CDVA was CDVA was equal to or better than preoperative levels in 98% of the ≤ 0.75D group and 95% of the > 0.75D group (*P* < 0.05). No significant overcorrection or undercorrection was observed. There were significant differences in vertical coma between preoperative and postoperative values in both groups (all *P* < 0.05), while there was no significant difference in other higher-order aberrations (*P* > 0.05).

**Conclusion:**

Ray-Tracing Guided-LASIK surgery is safe and effective. Compared with the preoperative BCVA, it can achieve better postoperative UDVA and effectively avoid the introduction of HOAs. In addition, for cases in which the MRSE difference between the two preoperative examination methods is ≥ 0.75, surgical design parameters adjustment can achieve ideal postoperative results.

## Introduction

Since the advent of refractive surgery, various surgical methods have emerged, including corneal laser surgery and intraocular lens implantation [10, 17]. Among these, Small incision lenticule extraction (SMILE) and laser-assisted in situ keratomileusis (LASIK) are the most widely used surgical methods for corneal refractive surgery [4, 21, 28]. Currently, the most commonly employed LASIK surgical techniques in clinical practice are topography-guided LASIK, wavefront-optimized LASIK, and Q-value-guided LASIK, all of which have demonstrated strong safety and efficacy in clinical applications [9, 15, 26]. Topography guided LASIK is designed for surgery by collecting information points from corneal topography maps, which has a significant effect on irregular astigmatism of the cornea [1, 25]. Wavefront optimized LASIK and Q-value guided LASIK modify surgical plans based on Wavefront and Q-value data, respectively [3, 18].To further enhance LASIK precision, new calculation methods and ablation profiles are constantly being applied to refractive surgery, particularly in combination with artificial intelligence (AI)(Jayadev & Shetty [14, 24]. The integration of AI in ophthalmic surgery has significantly enhanced the work efficiency of ophthalmic clinicians and is gradually advancing ophthalmic surgery toward precision medicine. In the case of combining LASIK with artificial intelligence, topography-guided LASIK planned using the Phorcides Analytical Engine showed good postoperative results [5]. Ray-Tracing Guided LASIK, which incorporates measurements based on ray tracing principles and artificial intelligence, is designed to help patients achieve improved visual quality.

Ray-Tracing Guided LASIK, based on Innoveyes ™ platform and Implemented through sightmap detection, can create a 3D eye model for the entire eye optical system to guide refractive surgery. This approach fully combines wavefront aberration (Hartmann-Shack Wavefront sensor), anterior and posterior corneal topography (Scheimpflug Imaging), the position of the anterior lens surface, and the axial length of the eye (Partial coherence interferometry). By using iterative algorithms, a realistic 3D eye model is constructed, allowing for the determination of an optimal corneal cutting contour based on big data and advanced algorithms. This technique was initially applied in ophthalmic clinical practice for lens-related surgeries and achieved good results for issues such as the calculation of artificial lenses [11, 23]. At present, ray tracing technology has been implemented in corneal refractive surgery using sightmaps. However, in these early stages of application, clinicians exercise caution and strictly control the indications for its use. Research results indicate varying degrees of differences between the sightmap and subjective refraction, with significant differences in individual patients, and there may be overcorrection or undercorrection after surgery [6]. Current research is mostly based on optometry differences within the range of ± 0.5D or ± 0.75D [7,15]. Scholars continue to debate whether adjustments should be made to spherical and cylindrical lenses when there is a significant difference between the results of subjective refraction and sightmap examinations. This issue warrants further investigation.

Based on the current issue, we are exploring whether manually adjusting the differences between sightmap and subjective refraction can achieve the same effect for patients with significant differences. Therefore, this study compared the postoperative safety and effectiveness in patients with a difference of > 0.75D between sightmap examination results and apparent optometry results with a difference of ≤ 0.75D. Additionally, it examined the feasibility of parameter adjustment for patients with significant differences.

## Materials and methods

### Study design

This prospective cohort study included patients who underwent ray-tracing guided myopic LASIK surgery at Liaoning Aier Eye Hospital (China) between July 2023 and December 2023, comparing clinical outcomes up to 6 months postoperatively. This study was approved by the Ethics Committee of Liaoning Aier Eye Hospital (Ethics Number: 2023-013-01) and complied with the Declaration of Helsinki.

### Patients

The inclusion criteria were as follows: Patients who voluntarily selected ray-tracing guided LASIK surgery after examination at our hospital; The patient met the surgical indications for ray-tracing guided LASIK and is able to undergo sightmap check; The patient is ≥ 18 years old, with stable refractive error for more than 1 year, no other eye diseases, no history of other eye surgeries, no contraindications for LASIK surgery, and has completed a 6-month follow-up after surgery.

The exclusion criteria were as follows: presence of other eye diseases, prior eye surgery or other contraindications for LASIK surgery, unsuccessful sightmap evaluation, and incomplete 6-month follow-up after surgery.

### Examination and surgical methods

All patients underwent comprehensive preoperative examinations, including subjective refraction, cycloplegic refraction, fundus examination, corneal topography examination, and high-order aberration (Tracey iTrace). In addition, measurements were obtained using a ray-tracing guided LASIK device (InnovEyes sightmap), and the data were exported to the WaveNet server (Alcon Laboratories, Inc.) and excimer laser (EX500, Alcon Laboratories, Inc.). All subjective refraction and InnovEyes sightmap detection results with equivalent spherical deviation exceeding ± 0.75D require nomogram adjustment. Treatment value and subjective refraction value in the 6.5 mm area were adjusted to reduce the equivalent spherical deviation to within ± 0.75D. The adjustment sequence is to confirm the axis position first, then adjust the cylindrical mirror to control the difference value of the cylindrical mirror within ± 0.75D, and finally adjust the spherical mirror to make the equivalent spherical mirror error less than ± 0.75D. All surgeries were performed by the same surgeon (XJ.F), and the surgical instruments used were the Wavelight FS200 (Alcon Laboratories, Inc.) and Wavelight EX500 (Alcon Laboratories, Inc.). The corneal flap thickness was set to 110 microns, the treatment area was 6.5 mm, and the ablation contour was generated using InnovEyes sightmap data.

### Postoperative assessment

The postoperative follow-up period was 6 months, and the main measurement indicators were UDVA, CDVA, MRSE, total HOAs (RMS total HOAs), and mean HOAs, including root mean square values for Zernike orders 3 to 6 (RMS 3 to 6). The Alpins vector analysis method was used to calculate the correction index (CI)astigmatism [8].

### Statistical analysis

All continuous variables were expressed as mean ± SD, Shapiro-Wilk and Komogorov-Smimov was used for the assessment of normality of continuous variables, and means were compared using Student’s t-test or Mann-Whitney U test for numerical values, and likelihood ratios and ategorical variables were compared using the chi-squared test, and statistical significance was considered at an alpha level of < 0.05. Statistical charts were drawn using Excel 2017 and GraphPad Prism10.

## Results

### Baseline characteristics

This study included 46 patients (89 eyes), of which 46 eyes had no nomogram adjustment (conventional group), and 43 eyes had nomogram adjustment (adjustment group). Baseline of the two groups of patients is shown in Table 1. The surgical parameters for 17 patients were binocular adjustment, 7 patients were monocular adjustment, 19 patients had no adjustment for both eyes, and 3 patients underwent monocular surgery.

**Table 1:** Patient’s preoperative baseline characteristics

Parameter	Total	Conventional group	Adjustment group
No. of eyes	89	46	43
Age (years)	25.01 ± 5.81	25.09 ± 5.63	24.94 ± 6.07
Gender(F/M)	23/22	14/13	12/12
Sphere(D)	-4.39 ± 1.53	-3.85 ± 1.54	-4.95 ± 1.30
Cylinder(D)	-0.89 ± 0.59	-0.86 ± 0.59	-0.93 ± 0.61
MRSE(D)	-4.83 ± 1.58	-4.28 ± 1.60	-5.41 ± 1.45
CDVA (logMAR)	-0.01 ± 0.11	0.00 ± 0.04	0.02 ± 0.15
CCT(µm)	544.50 ± 25.01	545.80 ± 28.71	543.20 ± 29.61

MRSE: manifest refraction spherical equivalent, UDVA: uncorrected distance visual acuity, CDVA: corrected distance visual acuity, CCT: Central corneal thickness, D: diopters

### Refractive outcomes

**Fig. 1:**
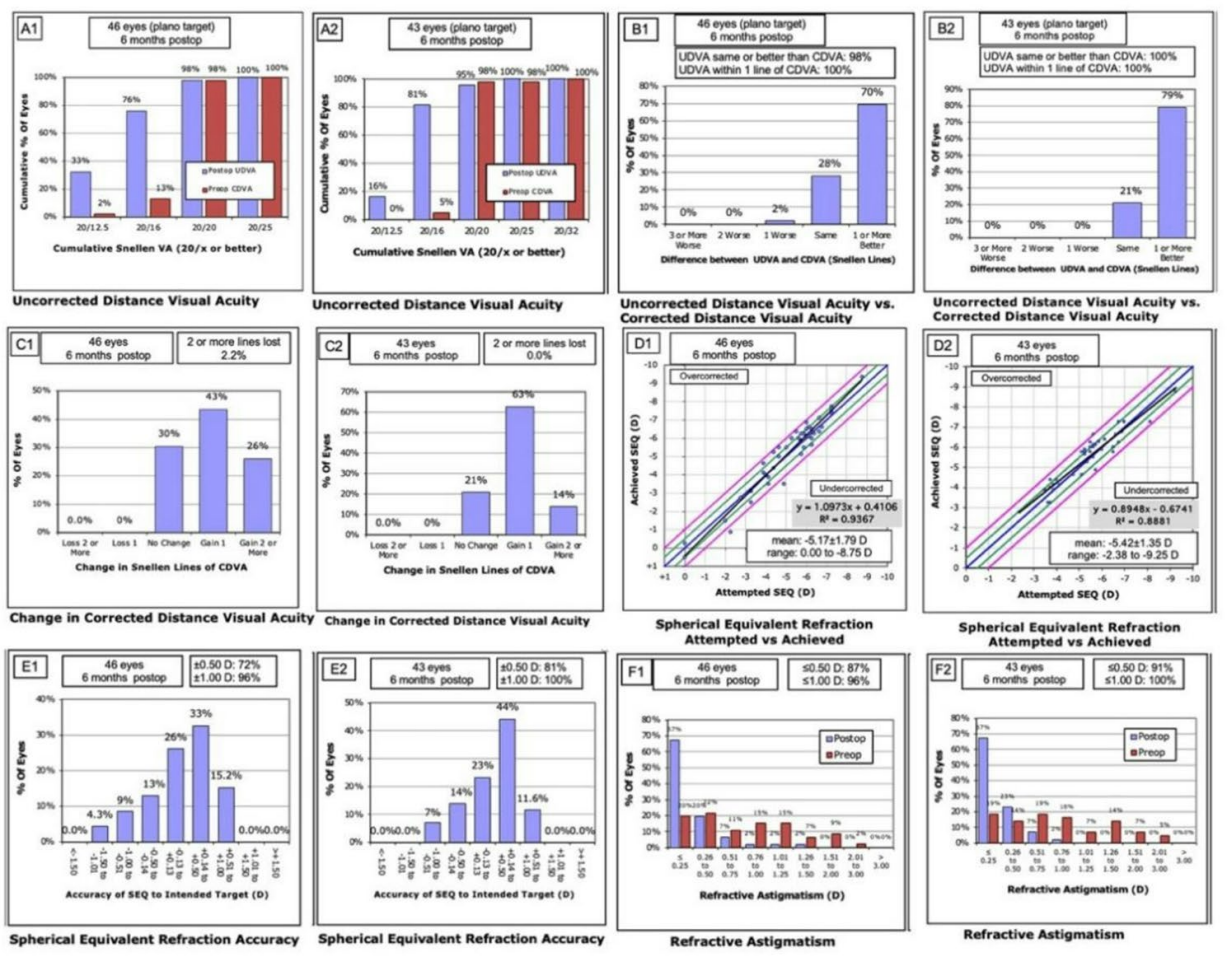
Postoperative refractive outcomes in the conventional and adjustment groups. **A1**: The CDVA outcomes of the conventional group. **A2**: The CDVA outcomes of the Adjustment group. **B1**: The UDVA vs. CDVA of the conventional group conventional group. **B2**: The UDVA vs. CDVA of the Adjustment group. **C1**: Change in CDVA of the conventional group. **C2**: Change in CDVA of the Adjustment group. **D1**: Attempted SEQ vs. Achieved SEQ of the conventional group. **D2**. Attempted SEQ vs. Achieved SEQ of Adjustment group. **E1**: Accuracy of SEQ to intended target of conventional group. **E2**: Accuracy of SEQ to intended target of Adjustment group. **F1**: Refractive Astigmation of conventional group. **F2**: Refractive Astigmation of Adjustment group

At the 6-month follow-up, 100% of patients in both groups achieved postoperative UDVA of 20/25 or better. The proportions of UDVA reaching 20/16 in the conventional group and adjustment group were 76% and 81%, respectively (Fig. 1A1, Fig. 1A2). The proportions of the conventional group and adjustment group with UDVA equal to or better than the preoperative CDVA were 98% and 95%, respectively (Fig. 1B1, Fig. 1B2). No patients in either group experienced a loss of 2 lines of CDVA, and no significant overcorrection or undercorrection was observed (Figure. C1-D2). The proportion of postoperative MRSE within ± 1.0D in the conventional group and adjustment group was 96% and 100%, respectively, while the proportion within ± 0.50D was 72% and 81%, respectively (Figure. 1E1, 1E2). The proportion of postoperative astigmatism ≤ 1.0D is 96% and 100%, respectively, and the proportion of astigmatism ≤ 0.5D is 87% and 91%, respectively (Figure. 1F1, 1F2). The above results showed no significant differences between the two groups (all *P* > 0.05).

### Astigmatism analysis

**Fig. 2:**
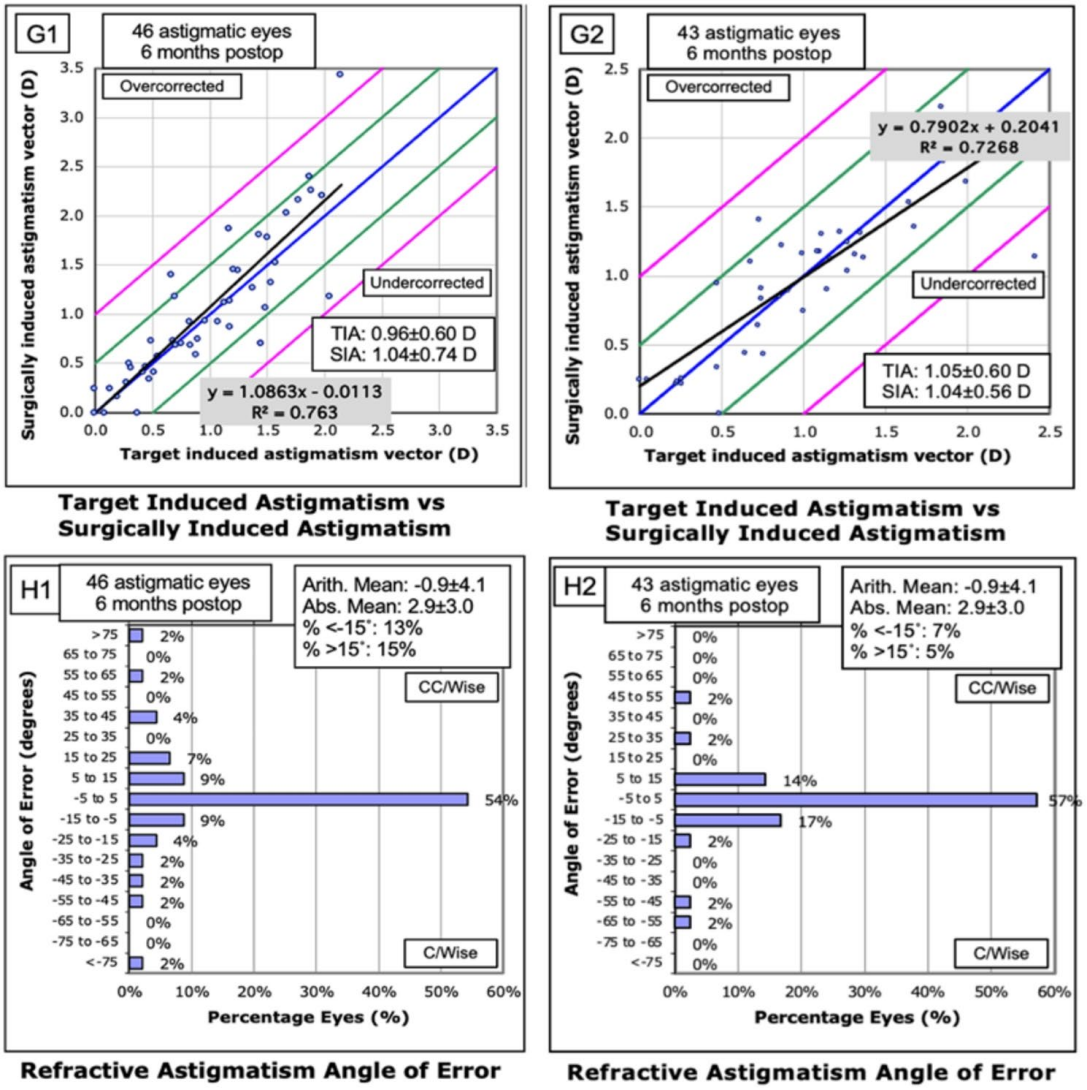
Postoperative astigmatism outcomes in the conventional and adjustment groups. **G1**: Target induced astigmatism vs. surgically induced astigmatism in the Conventional group. **G2**: Target induced astigmatism vs. surgically induced astigmatism in the Adjustment group. **H1**: Refractive astigmatism angle of error in the Conventional group. **H2**: Refractive astigmatism angle of error in the adjustment group

The target-induced astigmatism (TIA), surgically Induced astigmatism (SIA), difference vector (DV), and correction index (CI) results for the conventional and adjustment groups are shown in Table 2, and there were no significant differences between the two groups (all *P* > 0.05). The mean CI was 1.06 ± 0.40 in the conventional group and 1.01 ± 0.83 in the adjustment group, with no significant difference between the two groups (*P* = 0.54, Fig. 2G1, 2G2). The percentages of the angle of error (AE) within ± 15 °for the conventional and adjustment groups were 68% and 88%, respectively, and there were significant differences (*p* = 0.02, Fig. 2H1, 2H2).

**Table 2:** Outcomes of vector analysis via the comparison of the conventional group and adjustment group

	Conventional group	Adjustment group	*P* vaule
TIA	0.96 ± 0.60	1.05 ± 0.60	0.46(NS)
SIA	1.04 ± 0.74	1.04 ± 0.56	0.99(NS)
DV	0.48 ± 045	0.42 ± 0.61	0.97(NS)
CI	1.06 ± 0.40	1.01 ± 0.83	0.54(NS)

TIA: target-induced astigmatism; SIA: surgically induced astigmatism; DV: difference vector; CI: correction index; NS: No statistical difference

### Visual quality

**Fig. 3:**
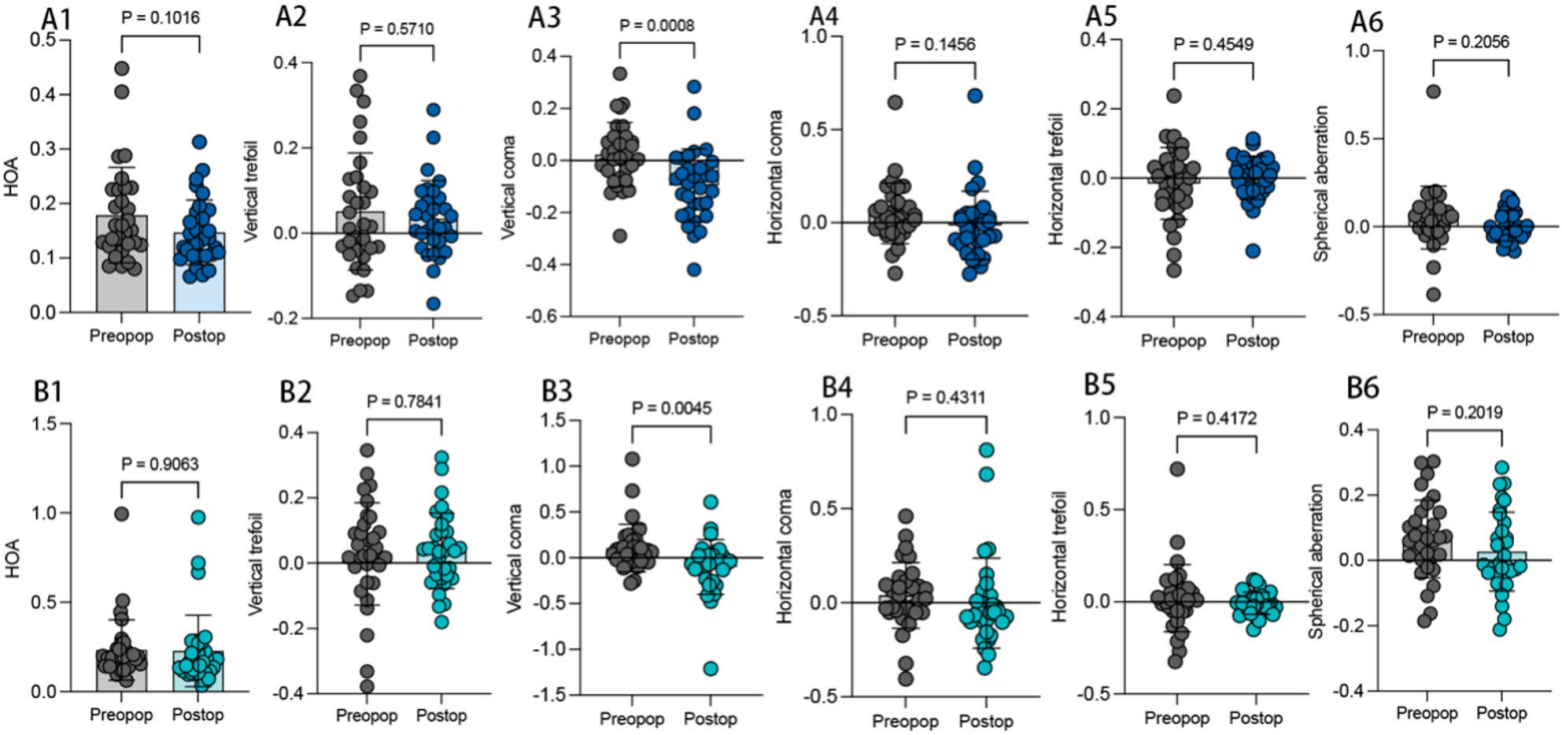
The vaule of HOAs. **A1**-**A6**: The Total HOA, vertical trefoil, vertical coma, horizontal coma, horizontal trefoil, Spherical aberration of Conventional group. **B1**-**B6**: The Total HOA, vertical trefoil, vertical coma, horizontal coma, horizontal trefoil, Spherical aberration of adjustment group

We analyzed the HOAs of patients 6 months after surgery and before surgery, including total HOA, vertical trefoil, vertical coma, horizontal coma, horizontal trefoil, Spherical aberration. We found that the vertical coma changed from positive to negative values, and there were significant differences between preoperative and postoperative values in both groups (*P* < 0.05); however, total HOAs, vertical trefoil, horizontal coma, horizontal trefoil, and spherical aberration were not significantly different (*P* > 0.05, Fig. 3A1-3B6).

## Discussion

Ray-tracing guided LASIK has been shown to be safe, effective, and predictable in several clinical studies. However, this surgical technology is not yet widely used in clinical practice, and specific aspects of the surgical design are still under investigation [6, 12, 15].Surgical indications are strictly controlled in most refractive surgery centers, with each patient undergoing rigorous screening. Patients with the result of SE and sightmap differences greater than ± 0.5D or ± 0.75D, they are usually refused to undergo Ray-Tracing Guided LASIK. Most patients in this group chose other types of LASIK surgery. The current low utilization rate of Ray-Tracing Guided LASIK results in a large number of patients not being able to enjoy the latest refractive surgery technology.

In our study, we adjusted the nomogram of patients with SEQ differences greater than ± 0.75D and compared the results of follow-up up to 6 months postoperatively with those of patients with equivalent spherical lenses and apparent optometry differences greater than ± 0.75D. Among patients with a difference of ≤ 0.75D between subjective refraction and InnovEyes sightmap results, 100% of patients achieved postoperative UDVA of 20/25, and the proportion of postoperative MRSE at ± 1.0D and ± 0.5D was 96% and 72%, respectively, which is similar to previously reported results [12]. Kanellopoulos et al., [15]. In our study, for the first time, parameter adjustments were made for patients with apparent differences of >0.75D between subjective refraction and InnovEyes sightmap results, and postoperative results were compared and analyzed with the group with smaller differences. The results showed that 100% of patients in both groups had postoperative UDVA of 20/25, and the proportion of postoperative MRSE at ± 1.0D and ± 0.5D was 100% and 81%, respectively, with no significant difference compared to the conventional group. In both groups, some patients did not achieve the target SEQ. Upon analysis, we found that the astigmatism axis in sightmap refraction differed significantly from that in subjective refraction. In practical applications, attention should be paid to the differences in the astigmatism axis, and appropriate adjustments should be made.

Most other refractive surgeries, including SMILE, topography-guided LASIK, and wavefront-guided LASIK, typically introduce varying degrees of HOAs after surgery, including spherical aberration and vertical coma, with a more significant increase in HOAs in patients with high myopia [2, 19, 20, 27, 29]. A study comparing Ray tracing guided LASIK and Q-Vaule guided LASIK suggests that the ray tracing group had significantly better postoperative corneal HOA and optical path difference [30]. Another study suggested that compared to Topography-Guided LASIK, Ray-Tracing-Guided LASIK on the InnovEys Sightmap platform has better postoperative visual outcomes [7]. The postoperative corneal HOAs and optical path difference were significantly better in the ray-tracing group. We found that there was no significant difference in total HOAs between preoperative and postoperative values in both groups(*P* >0.05), while there was a significant difference in vertical coma between preoperative and postoperative values in both groups(*P* < 0.05). This may be due to the upward shift of the laser cutting center during surgery, which introduced vertical coma. This may be related to the patient’s head position, eye rotation (especially uncompensated cyclokinesis), or the surgeon’s alignment habits. Studies have shown that vertical coma affects the reading ability of Roman characters, and even Air Force pilots exhibit lower levels of vertical coma than the general population [22]. Correcting vertical coma has been applied to enhance visual acuity in patients with keratoconus [16]. To improve reading ability in patients with intraocular lens implantation, demonstrating significant clinical benefits [13]. This also reminds us to pay attention to the center of the laser cutting during surgery.

## Conclusion

Ray tracking-guided LASIK surgery can achieve good refractive outcomes with a strong safety and efficacy profile. This approach minimizes the introduction of new high-order aberrations during the procedure at 6 months follow up, thereby supporting optimal postoperative visual quality in patients. In addition, the confirmation of the laser cutting center during surgery cannot be ignored.

### Limitations

This study has limitations, such as a small sample size, and insufficient follow-up time, and no subjective questionnaire survey was conducted before surgery. Therefore, we lacked robust evidence of postoperative visual quality. In the future, we plan to expand this study to a larger sample with long-term follow-up and design a comparative study with other LASIK techniques.

## Data Availability

The datasets used and/or analysed during the current study are available from the corresponding author on reasonable request.

## Abbreviations

LASIK: (Laser-Assisted in Situ Keratomileusis): A surgical procedure using a laser to reshape the cornea to correct refractive errors, including myopia, hyperopia and astigmatism to improve visual acuity
Ray-Tracing: A computational technique in ophthalmology used to model the path of light rays through the eye’s optical structures to enhance precision in corneal ablation during LASIK surgery
SEQ: Spherical Equivalent: A single value representing the overall refractive power of the eye, calculated from a combination of sphere and cylinder measurements in diopters, used to assess the extent of refractive error
Manifest refraction: A clinical measurement of a patient’s refractive error obtained through subjective refraction testing, used as a reference for prescription lenses or surgical planning
InnovEyes Sightmap: A diagnostic imaging tool utilizing 3D optical mapping to assess corneal and ocular surface structures, aiding in the accurate planning of customized LASIK treatments
HOAs: Higher-order aberrations: Complex irregularities in the eye’s optical system that affect visual quality, including aberrations like coma, spherical aberration, and trefoil, which are not corrected by standard lenses
UDVA: Uncorrected Distance Visual Acuity: A measure of a patient’s visual acuity without the aid of corrective lenses, commonly used as an outcome indicator in refractive surgery
CDVA: Corrected Distance Visual Acuity: Visual acuity measurement obtained with optimal corrective lenses, providing a baseline for evaluating improvements post-surgery
Vertical Coma: A specific type of higher-order aberration where light rays deviate asymmetrically across the vertical axis, often impacting visual clarity and quality
